# Collateral damage: the impact of the COVID-19 pandemic on the care of a patient with tuberculous neuroretinitis in Lagos, Nigeria

**DOI:** 10.11604/pamj.supp.2020.35.135.24691

**Published:** 2020-08-06

**Authors:** Temiloluwa Moyosoreoluwa Abikoye

**Affiliations:** 1 Metro Eye Centre, Lagos, Nigeria,; 2 Guinness Eye Centre, Lagos University Teaching Hospital, Lagos, Nigeria

**Keywords:** COVID-19, tuberculous neuroretinitis, ophthalmology, healthcare delivery

## Abstract

Tuberculous neuroretinis, a relatively rare manifestation of extra-pulmonary tuberculosis, is characterized by optic disc edema, peripapillary and macula swelling, with hard exudates forming a partial or complete 'macular star' While the disease may present a diagnostic challenge for Ophthalmologists, prognosis is usually good, with proper management. The Coronavirus Disease 2019 (COVID-19) pandemic has presented a healthcare delivery dilemma in many parts of the world, with poor accessibility to, and under-utilization of, important healthcare services by non-COVID-19-related cases. Herein is a report of a case of tuberculous neuroretinitis in Lagos, Nigeria, whose care was negatively impacted by the ongoing pandemic through the combined factors of the interruption of clinical services during the lockdown, patient avoidance of healthcare facilities and the absence of robust telehealth services. These all culminated in the delayed institution of therapy which may be responsible for the poor visual outcome of no-light-perception.

## Introduction

Tuberculosis (TB) is a communicable, disease caused by *Mycobacterium tuberculosis*, the diagnosis and treatment of which is a burden, particularly in developing countries, which bear the greater burden of the disease [1]. While the disease primarily affects the lungs, it may also affect the eye and as high as 60% of patients who present with intraocular tuberculosis (IOTB) do not have pulmonary TB [1]. This further complicates the diagnosis of the disease as there is no gold standard diagnostic test for IOTB [2]. Tuberculous optic neuropathy, a spectrum which encompasses neuroretinitis, papilledema, papillitis, optic neuritis, retrobulbar neuritis and optic nerve tubercles, may result from direct infection by the Mycobacteria or an associated hypersensitivity reaction [2]. Prompt diagnosis and management of the disease- an interdisciplinary approach by the Ophthalmologist and the Infectious Disease Specialist (IDS)- is recommended [2]. The treatment guideline recommended by the Centre of Disease Control and Prevention (CDC), American Thoracic Society, and the Infectious Diseases Society of America, is the use of the anti-TB quadruple therapy (rifampin, isoniazid, pyrazinamide, and ethambutol) for two months followed by a four to seven-month continuation phase typically consisting of rifampin and isoniazid [3]. The importance of the adjunctive use of low dose oral steroids for the reduction of inflammation in select cases of IOTB is documented, however, a significant beneficial effect on the final visual acuity has not been proven [2].

The Coronavirus Disease 2019 (COVID-19) pandemic is a global phenomenon that has greatly shaped the first half of the year 2020, with projected long-lasting effects across all sectors- notably the health sector [4]. While many emergency care services in 'hard-hit' locations have been overwhelmed by the number of patients plagued by the virus, many non-COVID-19-related medical services have been scaled back or shut down altogether [5]. The under-utilization of important medical services by patients with non-COVID-19-related urgent and emergent health needs is constituting an important problem for health care systems [6]. Patients delay presentation at hospitals despite life threatening symptoms and healthcare workers are having to deal with a 'trade-off between the patients' needs for procedures and the need to protect the caregivers from infection [7]. To aid healthcare systems with balancing the need to provide essential services, while minimizing risk to the caregivers, the CDC provided guidelines for delivery of non-COVID-19 health care during the pandemic [8]. One of the core components of this transformation of healthcare service delivery is the increased utilization of telemedicine for patient care. While the healthcare systems in developed countries may have been better poised to implement telemedicine at a larger scale, due to the prior widespread utilization of Electronic Medical Records (EMR), this advantage is not universal.

The COVID-19 pandemic amplified many preexisting deficiencies of the healthcare delivery system in Nigeria, notably, the infrastructure important for the establishment of effective tele-medical services. Most healthcare providers in the country utilize paper medical records and lack electronic patient referral systems [9]; referrals and consultation requests largely rely on the patients being the couriers of the request letters [10] and there is sparse, publicly available information of the contact details for healthcare providers. The combined effects of the reduced accessibility to clinical/laboratory services, insufficient infrastructure for effective telehealth service delivery and the difficulty in facilitating inter-practice medical consultations have negatively impacted health care delivery to, and the eventual outcomes of, patients with non-COVID-19-related health problems. Herein, the author reports a case of tuberculous neuroretinitis in Nigeria whose care, and outcome, was impacted by the ongoing pandemic.

## Patient and observation

A 21-year-old female presented with a one-month history of severe left eye pain, associated with headaches and a 2-week history of gradual reduction of vision. Her history was positive for unexplained weight loss of 2 years´ duration, remitting and relapsing suppurative cervical masses, loss of appetite, nausea and vomiting after meals. She had no history of low grade fevers, night sweats, rashes, exposure to persons with chronic cough or exposure to cats. She is a chronic severe peptic ulcer disease patient on medication. The patient recently, abruptly, relocated from the northern part of Nigeria, at the commencement of the COVID-19 pandemic lockdown, two months before presentation. She had previously sought medical care on account of the weight loss and neck masses. All investigations carried out were non-revealing, by her account. She did not possess copies of these results, a medical report or the contact information for her previous physician. She delayed presentation to an eye-care provider, at the onset of her ophthalmic symptoms, due to the temporary closure of most private eye hospitals during the first phase of lockdown. She did not present at any public eye-care emergency hospitals in order to avoid contact with crowds of people, as a preventive measure against contracting the COVID-19 virus.

**Figure 1:**
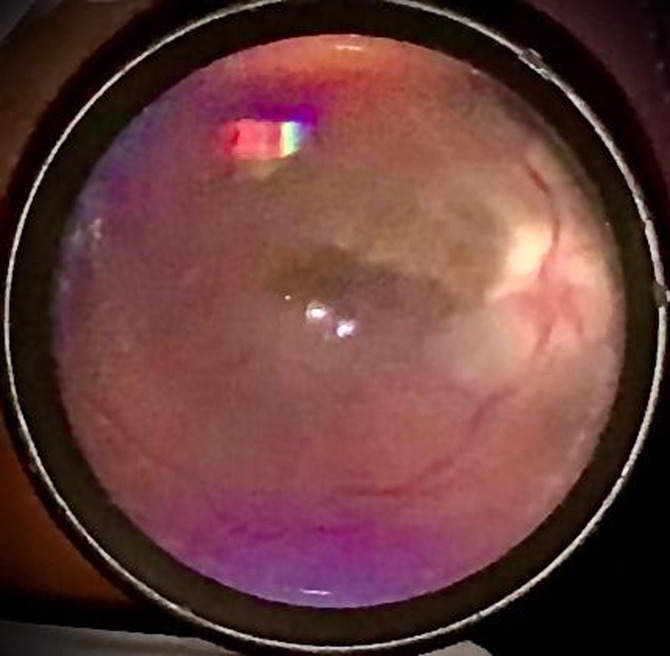
fundus photograph of the left optic eye, taken with an iPhone Xr mobile phone and 20D Volk^®^ lens, at initial presentation. Swelling of the optic nerve head, peripapillary area and macula seen. Artifacts from camera flashlight

On general physical examination, the patient was chronically ill looking and she had multiple, visibly enlarged cervical and supraclavicular lymph nodes bilaterally (each ~ 5 x 3cm). The left enlarged supraclavicular lymph node was ulcerative, with purulent discharge. Significant eye examination findings were best corrected visual acuity of 6/9 in the right eye and no perception of light (NPL) in the left eye. Extraocular motility was full in both eyes, however there was left eye pain with movement. The left pupil was dilated and unreactive to light. There was no sign of anterior segment or vitreous inflammation. Fundus examination showed remarkable optic disc and macular swelling, with a 'macular fan' hard exudates (lipid deposition). The right eye examination was normal. Multiple tests were ordered, including complete blood count and blood film, Flourescent Treponemal Antibody Absorption (FTA-ABS), Angiotensin Converting Enzyme (ACE), Lyme serology, TB-quantiferon gold-assay and Retroviral screening tests. Fundus photography and orbital B-scan tests were scheduled to be carried out peripherally as ocular investigations at our facility had been suspended. However, a quick baseline fundus photograph of the left optic nerve head was taken in the clinic, using the iPhone XR mobile phone and 20D Volk^®^lens (Figure 1). Empirical oral ciprofloxacin was commenced pending the return of the test results. A two-day follow-up appointment, for the review of the test results, was scheduled. Patient returned to the clinic after two weeks. Laboratory services had been disrupted by the pandemic lockdown and she chose not to return without the investigation results. Her TB-quantiferon gold assay result was positive while other test results were non-revealing. The ocular investigations had not been carried out. Ocular examination remained status quo.

At this visit, patient revealed that prior to the pandemic-lockdown; plans for a clinical trial of Anti-Tuberculosis-Therapy (ATT) had been instituted by her previous physician. These plans were disrupted by the onset of the lockdown and her subsequent unplanned relocation. The patient was urgently referred to an IDS, through her new primary care physician, with recommendations for prompt initiation of ATT. Oral steroid therapy was deferred till after the start of ATT. Clearance for the initiation of steroid therapy was requested from the IDS. Her history of severe chronic severe peptic ulcer disease was noted as a relative contraindication to the therapy. Ocular investigations were re-scheduled and a one-week follow up clinic appointment was scheduled. She presented for a third clinic visit after 4weeks. Quadruple therapy of ATT (Rifampicin, Isoniazid, Pyrazinamide and Ethambutol) had been instituted for four weeks. Patient declined to come in sooner, in her continued bid to reduce her risk for COVID-19 infection. She had also defaulted from the follow up appointment with the IDS. Her clinical appearance was much improved at this visit- appetite was restored, post-meal vomiting resolved, weight gain ~ 2.5kg and suppurative lymph nodes had healed. Visual acuity in the left eye was Hands-Motion with sluggish pupillary constriction to light observed. There was complete resolution of the optic nerve and retina swelling, with waxy disc pallor, residual hard exudates at the macula, macular hyperpigmentation and thinning.

**Figure 2:**
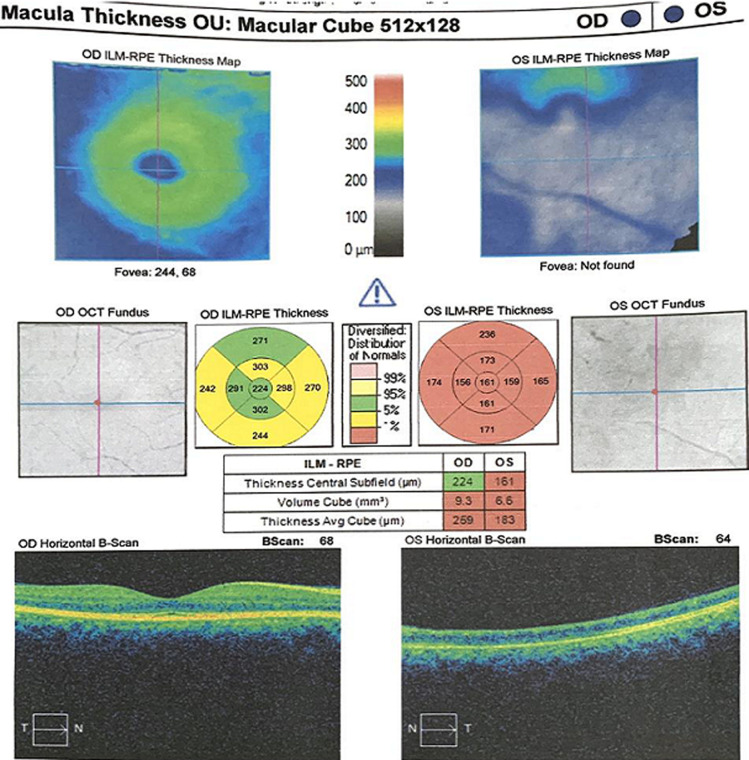
optical coherence tomography report, showing the macula thickness of both eyes after 5 weeks on ATT. There is marked thinning of the outer and inner retinal layers in the left eye, with loss of the foveal contour. There are no intraretinal or subretinal edemas in the left eye

**Figure 3:**
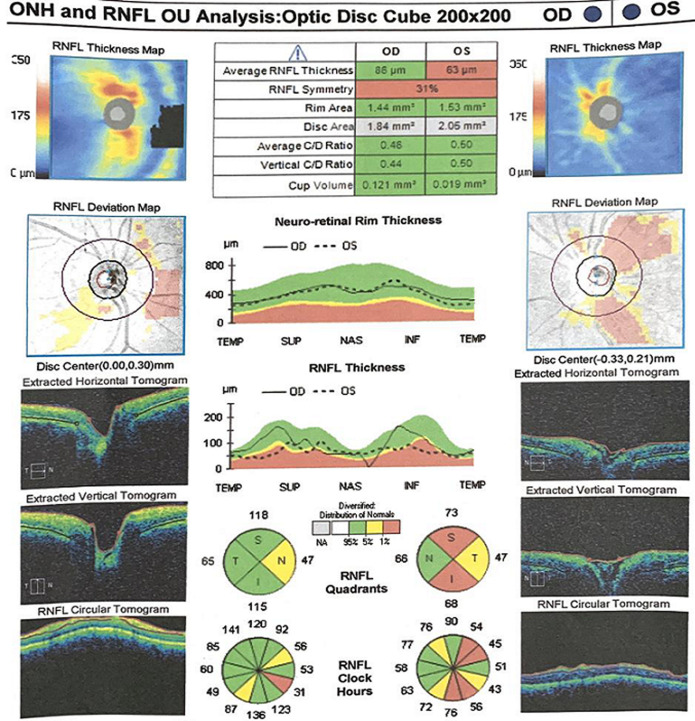
optical coherence tomography report, showing the optic nerve head and the retinal nerve fibre layer thickness of both eyes after 5 weeks on ATT. There is double-arcuate-shaped thinning of the peripapillary fibres in the left eye- representing macular fibres. Swelling of optic nerve head was completely resolved

Efforts to obtain the contact information for the IDS, for an interdisciplinary consult, proved abortive. A follow-up letter was sent, repeating the request for clearance for oral steroid therapy. The option of periocular steroid administration was discussed with the patient. A follow up clinic appointment of 1 week was scheduled. Patient returned 1 week later, as scheduled (5 weeks on ATT). Vision had deteriorated back to NPL. Fundal optical coherence tomography (OCT) testing was carried out at this visit. The findings in the left eye were severe thinning of the outer and inner retinal layers with loss of the foveal contour (Figure 2,Figure 3). Patient was still yet to present for a follow-up appointment with the IDS, due to the reasons stated earlier. A third letter was written, to the IDS, with an update about her condition and a request for an interdisciplinary phone consult. Independent efforts made by our clinic to obtain the name and contact information of the IDS have been yet unsuccessful. Weekly follow up consultations via video-conferencing have been set up. Patient was encouraged to keep her appointment with the IDS.

## Discussion

Tuberculous optic neuropathy is a relatively rare manifestation of extra-pulmonary TB, an important sign in areas of endemicity [1]. Tuberculous neuroretinitis is even less common. In the multinational study by Davis et al of 62 eyes, tuberculous neuroretinitis accounted for about 14.5% of cases of tuberculous optic neuropathy [2]. Similar to the case above, presentation is usually unilateral. Visual recovery from tuberculous neuroretinitis is good when diagnosis is prompt and proper management instituted, with only 10% of cases with vision < 6/60 after 12 months of follow up [2]. Although our patient has only been on ATT for 5 weeks, the NPL vision, as well as the fundal OCT findings, may be poor prognosis indicators. The care of our patient has been negatively impacted by the COVID-19 pandemic in multiple ways. Delayed patient presentation and unavailable clinical/laboratory services were important causes of the late diagnosis and management, as documented in other reports [6,7]. However, notable in this case is the reported disruption of the clinical trial of ATT, five weeks before the onset of her ocular symptoms. This therapeutic trial may have prevented the development of the neuroretinitis in its entirety. The interruption of ongoing clinical care was only worsened by the gap in the continuity of care. The announcement of the nationwide lockdown in Nigeria, 48 hours before its onset, precipitated the patient´s abrupt relocation to Lagos, without a referral letter or medical report. Patients can be poor medical historians, sometimes omitting relevant clinical information. Channels for direct communication between healthcare providers are important, particularly during a pandemic, where patients may be unexpectedly separated from their established care providers.

The establishment of telemedicine service is perhaps the most imperative, pressing need, by any healthcare delivery system at this time. For this patient, some of the appointments, by all the care providers, could have been carried out virtually, protecting the parties involved. While telemedicine is not well suited for performing a detailed physical or intraocular examination, useful information may be obtained from these consultations. The virtual consultations may have, furthermore, improved the patient´s compliance with requested in-clinic appointments, enhancing her care and maybe outcome. Finally, while the CDC has provided working guidelines for healthcare providers, centered on the use of telemedicine to reduce patient visits to the hospital, these may not alleviate the pandemic-induced-fear of presenting at healthcare facilities, by “high-risk” groups, when these visits are imperative. Our patient´s reluctance to visit the clinic was precautionary- due to her chronic ill health status. She is at a higher risk for adverse outcome, if infected by the COVID-19 virus. While our clinic has strictly adhered to the national guidelines for patient care during the pandemic, mobile healthcare delivery services may be necessary for the care of higher risk patients.

## Conclusion

This case report highlights the impact of the COVID-19 pandemic on the care of a patient with tuberculous neuroretinitis, an unrelated condition which usually has a good outcome. The institution of EMR and electronic referral systems, telehealth services, as well as the provision of mobile health-care delivery services, will be important, now more than ever, for ensuring accessibility to, and the continuity of, healthcare service delivery in these uncertain times. These important adjustments may help mitigate some of the collateral damages of the COVID-19 pandemic on healthcare as a whole.
